# Estimation of Dietary Amino Acid Intake and Independent Correlates of Skeletal Muscle Mass Index among Korean Adults

**DOI:** 10.3390/nu12041043

**Published:** 2020-04-10

**Authors:** Minjeong Chae, Hyoungsu Park, Kyong Park

**Affiliations:** 1 Department of Food and Nutrition, Yeungnam University, Gyeongsan, Gyeongbuk 38541, Korea; 2 Health & Nutrition R&D Group, Maeil Dairies Co., Ltd, Pyeongtaek, Gyeonggi 17714, Korea

**Keywords:** amino acid, dietary intake, skeletal muscle mass

## Abstract

The aim of this study was to develop a database to identify dietary amino acid intake levels, and to determine whether any amino acid groups were independently correlated with skeletal muscle mass index (SMI). We used data from the Korea National Health and Nutrition Examination Survey 2008–2011, and a total of 3292 participants aged 50–64 years were included in the analysis. Dietary data were obtained using the 24 h recall method. Data regarding dietary amino acid intake was assessed using the computer-aided nutritional analysis program 4.0 published by the Korean Nutrition Society. Multivariate linear regression analysis was used to identify independent correlates of SMI. The major food group that contributed the highest essential amino acid intake was grain and grain products (histidine 25.5%, isoleucine 43.9%, leucine 44.2%, methionine 31.0%, phenylalanine 44.8%, tryptophan 26.4%, and valine 50.8%). Higher SMI was independently associated with sex (men), lower age and body mass index, higher levels of physical activity, and a higher intake of energy and branched-chain amino acids. These results are expected to be used as a basis for developing dietary amino acid intake guidelines for Koreans.

## 1. Introduction

Amino acids are molecules that make up proteins and participate in processes such as neurotransmitter transport and biosynthesis [1,2]. In the human body, there are 20 amino acids, that are classified as either essential or non-essential based on nutritional requirements [3]. All amino acids contain an N-terminus, a C-terminus, and a central carbon atom with an attached side chain. The chemical properties of amino acids are dependent on the side chain present. Thus, they are divided into aromatic amino acids, branched-chain amino acids (BCAA), polar amino acids, and non-polar amino acids [3,4,5].

According to epidemiological studies, a sufficient intake of amino acids has a positive impact on hypertension, diabetes, and depression [6,7,8]. In particular, several studies have reported effects of amino acids on skeletal muscle maintenance [9,10]. However, current studies on the amino acid intake levels of Koreans are inadequate. Dietary Reference Intakes for Koreans are revised every five years, however the evidence for amino acid intake is insufficient, and thus follows standards established by other nations [11].

In 2020, the proportion of people in Korea aged 65 or over is 15.7%. This proportion is expected to increase to 21.4% by 2026 [12]. As sarcopenia results from age-related muscle decline, interest in preventing sarcopenia has increased [13], especially in Korean middle-aged and older people who have inadequate protein intake. Loss of muscle mass is caused by an imbalance in muscle protein synthesis and degradation that increases with age [14], and that can be influenced by lifestyle habits and is closely associated with diet [15]. Muscle mass can be reported as appendicular skeletal muscle mass (ASM). The skeletal muscle mass index (SMI) can be calculated as ASM/weight × 100 [15]. Scarcopenia is defined as two standard deviations below the mean SMI of healthy young individuals [16]. 

A cross-sectional study conducted in Norway showed a reduced plasma concentration of BCAA and lower intake of protein in participants with sarcopenia in an elderly population [17]. According to a recent clinical study in Japan, BCAA supplementation significantly increased muscle strength and muscle mass in adults over 65 years of age who had sarcopenia [18]. Another study using data from the United States National Health and Nutrition Examination Survey (NHANES), analyzed independent factors associated with SMI in participants over 50 years old [19]. The results showed that increased age and female sex were associated with lower SMI, while vigorous aerobic activity and non-obesity were positively correlated with SMI. In Korea, studies on the associations of physical activity [20], obesity [16], and protein intake levels [21] with sarcopenia have been conducted, but these studies were mainly limited to people over the age of 60 and gave insufficient consideration to overall correlates of SMI. In addition, since muscle loss initiates in middle age and accelerates thereafter [22], studies are necessary to identify independent dietary factors that can help to prevent sarcopenia.

The aim of this study was to develop a database of identified dietary amino acid intake levels, and to analyze independent correlates of SMI among adults aged 50–64 years, using nationally representative data from the Korea National Health and Nutrition Examination Survey (KNHANES). 

## 2. Materials and Methods 

### 2.1. Study Population 

The KNHANES is cross-sectional study conducted in Korea to generate representative and reliable statistics on health and nutritional status. Detailed KNHANES survey methods and protocols are described elsewhere [23]. Briefly, the KNHANES was first performed in 1998 and was initially conducted every three to four years, followed by the introduction of a rolling sample survey, which has been conducted annually, covering all weeks of the year since the fourth KNHANES (2007–2009). 

The present study used the KNHANES datasets from 2008–2011, in which data on muscle mass are available. The total number of respondents in KNHANES 2008–2011 was 37,753. We excluded participants who (1) were less than 50 or over 65 years of age; (2) had total energy intake less than 500 kcal or more than 5000 kcal per day or consumed more or less than they usually would on the day when their dietary intake was reported; (3) had a diagnosis of cancer; and (4) had missing values on ASM information (*n* = 34,461). A total of 3292 participants were included in the analysis. All participants provided written informed consent. The study followed the guidelines of the Declaration of Helsinki, and the study was approved by the Institutional Review Board of the Korea Centers for Disease Control and Prevention (approval number: 2008-04EXP-01-C 2009-01CON-03-2C 2010-02CON-21-C 2011-02CON-06-C).

### 2.2. Demographic and Lifestyle Information

Information on age, sex, income, and education level was collected via interview by trained investigators, while data on alcohol, smoking status, and physical activities were collected through self-report questionnaires. Household income was divided into quartiles based on monthly average household equalization income, and education level was divided into two groups: those who did not graduate from high school and those who graduated from high school or higher. Individuals were categorized as smokers or non-smokers based on current smoking status; alcohol consumption was classified as drinkers or non-drinkers. Physical activity was calculated from the number of days and hours of intense, moderate, or walking physical activity, which was then converted into metabolic equivalents of task (METs-h/week) using weekly physical activity time weighted by exercise intensity [24]. Subsequently, these values were categorized into tertiles of low-, medium-, and high-activity levels. 

### 2.3. Anthropometry and Dietary Assessment

Anthropometric data were assessed by trained medical staff. Body weight and height were measured in light clothing with shoes and socks removed. Body mass index (BMI) was calculated as weight in kilograms divided by height squared in meters. 

Dietary information was obtained using a single 24-h recall, in which a trained interviewer visited households and participants were asked to recall meal times, locations, and the quantity of food consumed. Food containers, food models, measuring cups, measuring spoons, a 30 cm ruler, and tape measures were used to assist in recall. The data was converted into individual foods using the food recipe database published by Korea Health Industry Development Institute [25], and nutrient intake was calculated using a food composition table developed by the Rural Development Administration of Korea [26].

To construct a dietary amino acid database, all food items from the 24-h recall data of people 50–64 years old who participated in the 2008–2011 KNHANES were screened. The Korea Nutrition Society’s Computer Aided Nutritional analysis program (CAN-Pro) 4.0 was used to calculate the dietary amino acid content of foods [27]. Moisture conversion factors developed by the Korea Centers for Disease Control and Prevention were applied to food items with different processing or preparation methods (boiled, raw, baked, or dried) [28]. For mixed foods, the amino acid content was calculated to reflect the food composition ratio. For processed foods, the information provided by the manufacturer was used to calculate the food content (e.g., the amount of powdered cream in coffee mix). If no specific product name existed, the commonly consumed Korean food was selected and substituted. Using this process, values from 2071 food items were estimated for the following 19 amino acids: alanine, arginine, aspartate, cysteine, glutamate, glycine, histidine, isoleucine, leucine, lysine, methionine, phenylalanine, proline, serine, taurine, threonine, tryptophan, tyrosine, and valine. For the statistical analysis, these were further classified into essential (histidine, isoleucine, leucine, lysine, methionine, phenylalanine, threonine, tryptophan, and valine), non-essential (alanine, arginine, aspartate, cysteine, glutamate, glycine, proline, serine, taurine, and tyrosine), branched-chain (isoleucine, leucine, and valine), aromatic (phenylalanine, tryptophan, and tyrosine), polar (cysteine, glutamate, serine, threonine, and tyrosine), and non-polar (alanine, glycine, isoleucine, leucine, methionine, phenylalanine, proline, tryptophan, and valine) amino acids [4,5].

### 2.4. Calculation of Skeletal Muscle Mass Index (SMI)

SMI was calculated according to the criteria suggested by Janssen et al. [29]. The ASM was calculated by excluding the weight of bone and fat in the arms and legs using a value measured by dual-energy X-ray absorptiometry. SMI was estimated as ASM/weight × 100.

### 2.5. Statistical Analysis

Nutrient intake levels were evaluated based on the estimated average requirement (EAR) and recommended nutrient intake (RNI) of the 2015 Dietary Reference Intake values for Koreans [11]. All food items were classified into 18 food groups, based on the criteria of the KNHANES. Food groups included grains and grain products, potatoes and potato products, sugars and sugar products, legumes and legume products, seed and seed products, vegetables, mushrooms, fruit, meats and meat products, egg, fish and shellfish, seaweeds, milk and dairy products, oils and fats, beverages and alcohols, seasonings, prepared foods, and other products. All statistical analyses were performed considering the KNHANES’ complex sampling design (stratification variables, primary sampling unit), and were conducted by reflecting the proper weighting of the survey segments (health interview, health examination, and nutrition survey). Multivariate linear regression analysis was performed to identify independent correlates of SMI, using various demographic and lifestyle factors (sex, age, education level, BMI, smoking status, household income, physical activity level, and alcohol consumption), as well as intake levels of total energy, essential amino acids, non-essential amino acids, aromatic amino acids, polar amino acids and non-polar amino acids as covariates. All statistical analyses were performed using Statistical Analysis System ver. 9.4 (SAS Institute, Cary, NC, USA). A significance level of α = 0.05 using two-tailed tests was considered statistically significant.

## 3. Results

General characteristics of the participants according to sex are shown in Table 1. In men and women, BMI (kg/m^2^) was 24.0 kg/m^2^ and 24.3 kg/m^2^, and the proportion of smokers was 39.3% and 3.6%, respectively. In addition, more than half of the participants were alcohol drinkers. The average skeletal muscle index levels were 31.9 ± 0.1% in men and 24.8 ± 0.1% in women. 

Dietary intakes of essential amino acids, as percentages of the EAR and RNI from the Dietary Reference Intakes for Koreans, are presented in Table 2. For all essential amino acid intake, men consumed higher percentages of the EAR and RNI than women (all, *p* < 0.001). Intakes of lysine, methionine, and phenylalanine were relatively lower than other amino acids, showing 87.7%, 85.3%, and 81.7% of the RNI for men and 74.3%, 73.9%, and 73.7% of the RNI for women. 

The top five food sources of amino acid intake are shown in Table 3. Commonly included among the top five food sources were grains and grain products, fish and shellfish, meats and meat products, legumes and legume products and vegetables. The major food group that contributed to essential amino acid intake was grain and grain products (histidine 25.5%, isoleucine 43.9%, leucine 44.2%, methionine 31.0%, phenylalanine 44.8%, tryptophan 26.4%, and valine 50.8%), which was 8%–39% higher than the next highest contributors of fish and shellfish, and meats and meat products. For lysine and threonine, the contribution from fish and shellfish were the highest at 27.7% and 21.2%, respectively. 

Table 4 presents multivariable-adjusted linear regression analyses for each of the identified amino acid groups and non-dietary factors for SMI. Sex, age, BMI, physical activity level, and energy intake were independent correlates of SMI. Men had a higher SMI than women (*p* < 0.001), and greater age (*p* < 0.001) and BMI (*p* < 0.001) were associated with lower SMI. In addition, more physical activity resulted in higher SMI (*p* < 0.001), and participants with a higher energy intake had higher SMI (*p* < 0.001). Among the amino acid groups, BCAA intake was positively associated with SMI (*p* = 0.01). On the other hand, education level (*p* = 0.12), smoking status (*p* = 0.16), alcohol consumption (*p* = 0.55), essential amino acid (*p* = 0.59), non-essential amino acid (*p* = 0.68), aromatic amino acid (*p* = 0.37), polar amino acid (*p* = 0.88), and non-polar amino acid intakes (*p* = 0.23) showed no significant correlation with SMI.

## 4. Discussion

Using data from KNHANES 2008–2011, this study established a database to identify dietary amino acid intake levels and their major food sources. Additionally, independent correlates of SMI were identified. In both men and women, essential amino acids methionine, lysine, and phenylalanine intakes were less than the RNI. The major food group contributing to essential amino acid intake was grain and grain products. SMI was independently associated with sex, age, BMI, physical activity, and intakes of energy and BCAA. 

In this population, intake of lysine, methionine, and phenylalanine in both men and women did not meet recommended levels. The major sources of these amino acids were grain and grain products, and this may be due to Korean vegetable protein-based dietary habits. A previous study that estimated dietary protein intake for people over 60 years of age using the KNHANES 2013–2014 found that the main source of protein was plant foods such as rice, with only one third of total protein intake coming from animal sources [30]. In addition, about half of the participants included in the study did not reach the RNI for protein, indicating that the quantity and quality of protein intake was inadequate. On the other hand, an NHANES in Japan found that participants aged 30–64 consumed approximately 50% of their total protein intake from animal foods, with their main sources being fish and meat [31]. The intake levels of the essential amino acids in men and women, respectively, were methionine 2.4 and 2.0 g/day, lysine 6.7 and 5.7 g/day, and valine 5.2 and 4.5 g/day; all of which were 2–4 g/day higher than the amino acid intakes in our study. In particular, methionine, lysine, and phenylalanine are found in greater amounts in animal proteins [32,33]. Therefore, the recommended intake of these amino acids would not have been reached due to the dietary habits of middle-aged Koreans who mainly consume plant foods, such as grains. Plant foods are also limited to amino acid compositions that lack one or more essential amino acids [34,35]. Therefore, in order to have a positive effect on muscle protein anabolism, it is recommended that high-quality amino acids are consumed through meat and dairy products that have high biological value [35,36].

In the multivariable-adjusted analyses, males had higher SMI, whereas age was associated with lower SMI. Skeletal muscle mass starts to decrease gradually from the 50 s [37], particularly in women, as estrogen is rapidly reduced due to menopause, which results in loss of skeletal muscle and a steeper decline in function compared to men [38]. Also, according to the New Mexico Elder Health Survey, the prevalence of sarcopenia under age 70 was higher in women, at 23–24% and 13–16% in women and men, respectively [39]. Another explanation is that males have increased muscle mass during pubertal development due to elevated circulating levels of testosterone [40]. Testosterone has a greater anabolic effect than estrogen [40,41], thus resulting in greater muscle mass in adulthood [22]. As a result, muscle strength and absolute power are greater for males than females because men typically start with a higher baseline value [42].

In this study, we showed an inverse association between BMI and SMI. This may be explained by the level of inflammation. A decrease in muscle mass and muscle strength during aging reduces physical activity [43]. This reduction in overall energy expenditure results in increased numbers of abdominal adipocytes, which secrete pro-inflammatory cytokines, which then increase levels of C-reactive protein and interleukin-6 [44,45]. In a cross-sectional study in the United Kingdom, the negative correlation between interleukin-8 and muscle strength/quality was observed in individuals with high adipose [46]. Consequently, obesity can increase pro-inflammatory cytokines, reduce protein synthesis, and promote degradation of myofibrillar proteins, leading to muscle wasting [47]. 

Physical activity increases a large number of mitochondrial biogenesis-related gene expressions [48]. Increased numbers of mitochondria could result in an enhanced adenosine triphosphate supply [49]. In a meta-analysis of the association between physical activity and sarcopenia, there was a 55% lower prevalence of sarcopenia in individuals performing high levels of physical activity [50]. It has also been shown that exercising with proper protein intake activates the mammalian target of rapamycin complex 1 (mTORC1) signaling that stimulates muscle protein synthesis and prevents muscle loss [51]. In a cohort study of middle-aged participants, higher protein intake combined with a higher physical activity level was associated with the preservation of muscle mass [52]. In a study in Australia, 100 women aged 60 to 90 were allocated into a group that consumed 160 g of red meat and performed resistance training or to a group that consumed pasta and rice and performed restricted exercise [53]. Results showed that there was a significant gain of 0.5 kg of lean tissue mass in the group that consumed red meat and performed resistance training.

In this study, participants with a higher energy intake had higher SMI, which is consistent with previous results [54,55]. Inadequate nutrition is a risk factor for sarcopenia [56], which can result in malnutrition if sufficient energy intake is not achieved due to digestive and physiological deterioration during aging. This can exacerbate the loss of skeletal muscle [54]. In a study that investigated the association between energy intake and the sarcopenia index (calculated by dividing the amount of ASM by the BMI and multiplying by 100) in Koreans over 30 years of age, total energy intake was found to be positively associated with the sarcopenia index [55].

Among the amino acid groups, BCAA intake levels were positively associated with SMI. The BCAA are leucine, isoleucine, and valine. Leucine regulates protein synthesis and transcription through activation of mTORC1 [57], which inhibits protein degradation and promotes synthesis [52,58]. A prospective study investigating the relationship between dietary intake of leucine and lean body mass change over six years in people aged 35–65 found that a greater dietary intake of leucine was associated with greater retention of lean body mass [9]. In addition, in a clinical study conducted in the United States, 20 healthy people in their 60s or older were given whey protein or 3 g leucine-enriched whey protein [10]. Ingestion of the leucine-enriched whey protein resulted in a larger muscle protein synthesis rate, which was higher when combined with exercise. A meta-analysis of clinical studies also showed an increase in net lean mass gains of 0.28%/week and in muscle strength of 1.4%/week with supplementation of beta-hydroxy beta-methylbutyrate (HMB), a metabolic form of leucine [59]. HMB increases lean body mass and muscle strength [60] and reduces exercise-induced muscle damage [59]. 

The current study has several limitations. First, dietary intake in this study may not reflect a person’s usual diet since it was calculated from a 24 h recall. This method could have resulted in misclassification. In order to minimize this limitation, we excluded the data of participants who consumed more or less than their usual diet on the day when their dietary intake was reported. In addition, nutrition surveys were conducted by trained interviewers using standardized protocols. Quality control and operational support were also conducted [61]. Second, levels of amino acid intake may have been underestimated, as there may be foods with amino acid content values that are unaccounted for in CAN-Pro. However, to compensate for this limitation, we included more food items that had different processing or preparation methods (e.g., dried vs. fresh) by applying the conversion factor provided by KNHANES. In addition, due to lack of information regarding the dietary supplements, amino acid intake through supplementation could not be considered. Third, the KNHANES is a cross-sectional study and, therefore, it is difficult to distinguish whether the identified independent correlates affected muscle loss or whether muscle loss affected identified correlates. Despite these limitations, this study is the first to analyze amino acid intake levels through the development of a dietary amino acid database, and to assess independent correlates of SMI in a middle-aged Korean population. We expect that these results will be useful in establishing the recommended nutrient intake of dietary amino acids, and in health policies to prevent sarcopenia. 

## 5. Conclusions

In conclusion, dietary methionine, lysine, and phenylalanine intakes in middle-aged Koreans were less than the RNI. SMI was higher in men than women, and age and BMI had an inverse correlation with SMI. Physical activity had a positive correlation with SMI, and there were positive correlations between intakes of energy and BCAA and SMI. These study results will be used to establish the recommended nutrient intake of dietary amino acids and in health policies for the prevention of sarcopenia. In future studies, prospective cohort or clinical studies should be conducted in elderly Koreans to consider the effects of obesity, age, resistance, and aerobic exercise on muscle synthesis.

## Figures and Tables

**Table 1:** Characteristics of participants according to sex.

Characteristics	Men	Women
	*N* = 1424	*N* = 1868
**Age (years)**	57.1 ± 0.1	56.7 ± 0.1
**Education level**		
Less than high school graduation	710 (50.1)	1304 (70.3)
High school graduation or higher	708 (49.9)	550 (29.7)
**Body mass index (kg/m^2^)**	24.0 ± 0.1	24.3 ± 0.1
**Smoking status**		
Non-smokers	862 (60.7)	1794 (96.5)
Smoker	559 (39.3)	66 (3.6)
**Household income**		
Low	343 (24.3)	466 (25.2)
Mid-low	377 (26.7)	459 (24.8)
Mid-high	355 (25.1)	483 (26.1)
High	338 (23.9)	441 (23.9)
**Physical activity level (METs-h/wk) ^1^**		
Low	401 (28.3)	683 (36.8)
Middle	468 (33.0)	631 (34.0)
High	550 (38.8)	541 (29.2)
**Alcohol consumption (yes)**		
Non-drinker	260 (18.4)	808 (43.6)
Drinker	1157 (81.7)	1045 (56.4)
**Skeletal muscle index (%)**	31.9 ± 0.1	24.8 ± 0.1

Values are mean ± standard error or *n* (%). ^1^ Physical activity was categorized into three groups, according to tertile of metabolic equivalents (METs)-hours/week.

**Table 2:** Essential amino acid intake as percentage of KDRIs by sex.

	EAR			RNI		
	Men	Women		Men	Women	
	*N* = 1424	*N* = 1868	*p*	*N* = 1424	*N* = 1868	*p*
**Essential amino acid (%)**						
Histidine	252.3 ± 3.6	209.5 ± 3.1	<0.001	196.2 ± 2.9	179.5 ± 2.6	<0.001
Isoleucine	276.4 ± 3.7	253.3 ± 3.2	<0.001	230.3 ± 3.0	202.6 ± 2.6	<0.001
Leucine	210.5 ± 2.8	188.2 ± 2.5	<0.001	171.6 ± 2.3	153.9 ± 2.0	<0.001
Lysine	111.6 ± 2.2	95.0 ± 1.9 *	<0.001	87.7 ± 1.7 *	74.3 ± 1.5 *	<0.001
Methionine	102.3 ± 1.9	92.3 ± 1.7 *	<0.001	85.3 ± 1.5 *	73.9 ± 1.3 *	<0.001
Phenylalanine	100.6 ± 1.3	91.2 ± 1.1 *	<0.001	81.7 ± 1.0 *	73.7 ± 0.9 *	<0.001
Threonine	170.5 ± 3.0	151.7 ± 2.6	<0.001	131.1 ± 2.3	121.4 ± 2.0	<0.001
Tryptophan	264.0 ± 3.6	195.1 ± 3.1	<0.001	176.0 ± 2.4	130.1 ± 2.1	<0.001
Valine	306.2 ± 3.5	272.4 ± 3.0	<0.001	245.0 ± 2.9	227.0 ± 2.5	<0.001

KDRIs, Dietary Reference Intakes for Koreans; EAR, estimated average requirement; RNI, recommended nutrient intake. Values are mean ± standard error. * Intake levels of the marked amino acids are lower than EAR or RNI.

**Table 3:** Food groups contributing to the essential amino acid intake.

Amino Acids	Contribution (%)	Intake (g/day)	Food Groups
Histidine	25.5	0.38 ± 0.2	Grain and grain products
	17.8	0.54 ± 0.6	Meat and meat products
	17.3	0.34 ± 0.6	Fish and shellfish
	13.0	0.19 ± 0.1	Vegetables
	6.5	0.14 ± 0.2	Legumes and legume products
Isoleucine	43.9	1.03 ± 0.5	Grain and grain products
	14.2	0.44 ± 0.9	Fish and shellfish
	12.8	0.62 ± 0.7	Meat and meat products
	8.1	0.28 ± 0.3	Legumes and legume products
	7.0	0.16 ± 0.1	Vegetables
Leucine	44.2	1.74 ± 0.7	Grain and grain products
	14.5	0.76 ± 1.6	Fish and shellfish
	13.7	1.11 ± 1.3	Meat and meat products
	8.7	0.50 ± 0.5	Legumes and legume products
	6.2	0.24 ± 0.2	Vegetables
Lysine	27.7	0.75 ± 1.3	Fish and shellfish
	25.6	1.08 ± 1.2	Meat and meat products
	13.1	0.27 ± 0.2	Grain and grain products
	11.7	0.35 ± 0.4	Legumes and legume products
	7.1	0.14 ± 0.1	Vegetables
Methionine	31.0	0.27 ± 0.1	Grain and grain products
	24.8	0.29 ± 0.5	Fish and shellfish
	18.1	0.32 ± 0.4	Meat and meat products
	5.3	0.07 ± 0.1	Legumes and legume products
	5.2	0.04 ± 0.1	Vegetables
Phenylalanine	44.8	1.00 ± 0.4	Grain and grain products
	13.1	0.39 ± 0.8	Fish and shellfish
	12.2	0.56 ± 0.6	Meat and meat products
	9.6	0.31 ± 0.3	Legumes and legume products
	7.0	0.16 ± 0.1	Vegetables
Threonine	21.2	0.41 ± 0.7	Fish and shellfish
	20.8	0.30 ± 0.2	Grain and grain products
	20.0	0.59 ± 0.7	Meat and meat products
	12.3	0.26 ± 0.3	Legumes and legume products
	9.2	0.13 ± 0.1	Vegetables
Tryptophan	26.4	0.12 ± 0.1	Grain and grain products
	19.6	0.12 ± 0.2	Fish and shellfish
	14.0	0.15 ± 0.2	Meat and meat products
	11.5	0.08 ± 0.1	Legumes and legume products
	9.8	0.04 ± 0.1	Vegetables
Valine	50.8	1.60 ± 0.7	Grain and grain products
	11.5	0.48 ± 0.9	Fish and shellfish
	11.0	0.72 ± 0.8	Meat and meat products
	7.5	0.23 ± 0.2	Vegetables
	6.4	0.30 ± 0.4	Legumes and legume products

Mean ± standard deviation.

**Table 4:** Amino acid groups and non-dietary correlates for skeletal muscle mass index.

Variables	β-Coefficient	*p*
**Sex, men**	6.99	<0.001
**Age (years)**	−0.05	<0.001
**Education level**		
High school graduation or higher	−0.19	0.12
**Body mass index (kg/m^2^)**	−0.41	<0.001
**Smoking status**		
Smoker	−0.20	0.16
**Household income**		
High	0.06	0.77
Mid-high	0.15	0.42
Mid-low	0.08	0.62
**Physical activity level (METs-h/week) ^1^**		
High	0.58	<0.001
Middle	0.77	0.003
**Alcohol consumption (yes)**	0.07	0.55
**Energy intake (kcal/day)**	0.0005	<0.001
**Essential amino acid intake (g/day) ^2^**	−0.04	0.59
**Non-essential amino acid intake (g/day) ^3^**	0.03	0.68
**Branched-chain amino acid intake (g/day) ^4^**	0.31	0.01
**Aromatic amino acid intake (g/day) ^5^**	−0.24	0.37
**Polar amino acid intake (g/day) ^6^**	0.01	0.88
**Non-polar amino acid intake (g/day) ^7^**	−0.10	0.23

Multivariable-adjusted findings including each of the presented variables. ^1^ Physical activity was categorized into three groups, according to tertile of metabolic equivalents (MET)-hours/week. ^2^ Essential amino acids: histidine, isoleucine, leucine, lysine, methionine, phenylalanine, threonine, tryptophan, and valine. ^3^ Non-essential amino acids: alanine, arginine, aspartate, cysteine, glutamate, glycine, proline, serine, taurine, and tyrosine. ^4^ Branched-chain amino acids: isoleucine, leucine, and valine. ^5^ Aromatic amino acids: phenylalanine, tryptophan, and tyrosine. ^6^ Polar amino acids: cysteine, glutamate, serine, threonine, and tyrosine. ^7^ Non-polar amino acids: alanine, glycine, isoleucine, leucine, methionine, phenylalanine, proline, tryptophan, and valine.
